# Improving the magnet alignment of undulator systems by laser interferometer

**DOI:** 10.1107/S1600577522001199

**Published:** 2022-03-14

**Authors:** Saif Mohd Khan, G. Mishra

**Affiliations:** a Insertion Device and Laser Instrumentation Laboratory (IDLI), Devi Ahilya Vishwavidyalay, Indore, Madhya Pradesh 452001, India

**Keywords:** undulator, misalignment, laser interferometer

## Abstract

The issue of intrinsic-type misalignment errors arising from angular offsets between magnets in an undulator is addressed. A random tilt of the magnets or poles generates undesirable magnetic field components in both transverse and longitudinal directions and gives rise to errors in period lengths and amplitudes. These localized errors are carried to the entire undulator segments and are a cause of concern for precision field integral and phase error estimates. A laser interferometer has been designed to read the offsets and to fix the magnets to minimize the offsets.

## Abstract 2

A laser interferometer has been developed to read and fix magnet misalignments in an undulator.

## 1. Introduction

**Figure 1:**
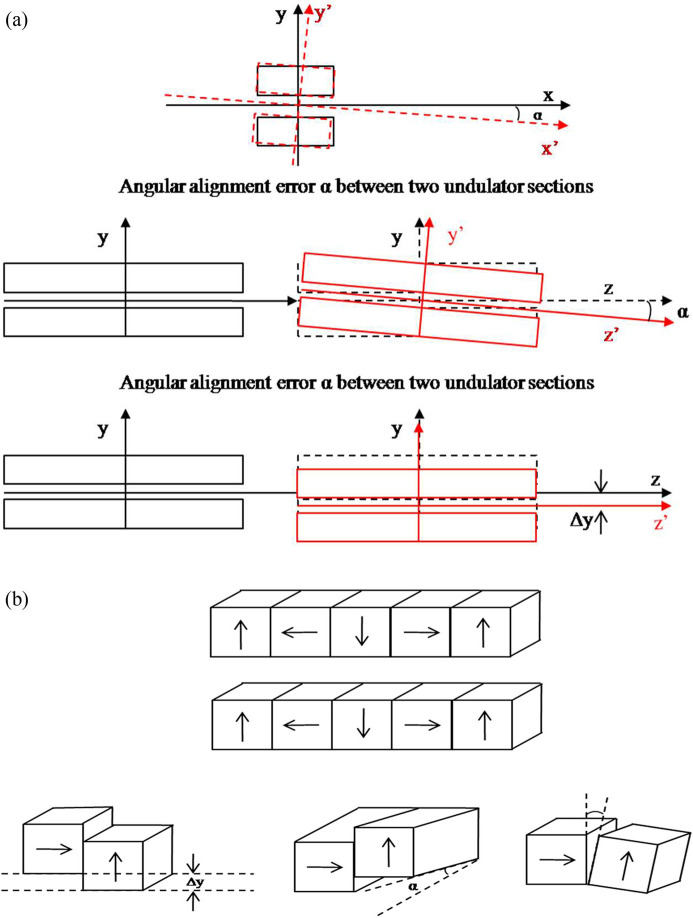
(*a*) Extrinsic misalignment. (*b*) Intrinsic misalignment.

Undulator technology (Takashi, 2015 ▸; Pflueger, 2016 ▸) has emerged as an important tool in light-source (Hwang *et al.*, 2011 ▸; Couprie, 2014 ▸), free-electron laser (Couprie, 2014 ▸; Jaeschke & Khan, 2020 ▸; Couprie, 2013 ▸; Huang *et al.*, 2021 ▸; Mishra & Sharma, 2020 ▸) and inverse free-electron laser accelerator (Duris *et al.*, 2012 ▸) applications. An undulator is a periodic arrangement of dipole magnets in the Halbach field configuration that produces a sinusoidal magnetic field. In a light source a relativistic electron beam propagates in a sinusoidal path along the undulator length according to the Lorentz force law and produces synchrotron radiation. The undulator facilitates the exchange of energy between the electron and a co-propagating laser, and the scheme operates as a free-electron laser or an inverse free-electron laser accelerator. The latest developments in undulator technology introduce several upgraded features to the free-electron laser and inverse free-electron laser accelerator. The free-electron laser today operates with a planar undulator, helical undulator, bi-period undulator, APPLE-type undulator, Delta undulator and Knot undulator. The inverse free-electron laser accelerator provides accelerating gradients of the order of GeV m^−1^ as compared with conventional RF accelerators with accelerating gradients of typically ∼100 MeV m^−1^. In practice, the undulator field quality is limited by the presence of several undesirable factors giving rise to undulator errors. The errors are mainly categorized as intrinsic or extrinsic type. A typical undulator for X-ray free-electron lasers is several meters long, and a long undulator is realized through several segmented undulator sections with drift sections in between. The performance of the free-electron laser depends on the alignment of each segment. A good longitudinal alignment between the segments gives a good phase matching between the radiation fields emitted from each segment. Position and angular offsets between the segments giving rise to misalignments between the segments are classified as extrinsic-type errors [Fig. 1 ▸(*a*)] (Reiche, 2009 ▸; Tanaka *et al.*, 2002 ▸; Mishra *et al.*, 2015 ▸). The undulator period is made up of four magnets and each segment undulator contains several undulator periods. The individual magnet’s position–angle offset and residual field mismatch between magnets gives rise to intrinsic-type undulator errors. Incorrect clamping of the magnets at both ends, upshifts/downshifts of the magnets, and tilts of the magnets along the undulator length give rise to intrinsic-type undulator errors [Fig. 1 ▸(*b*)]. These errors affect the performance of free-electron lasers in two ways – reduced transverse overlap and longitudinal desynchronization. The lack of straightness in the electron trajectory is a cause of reduced transverse overlapping between the radiation and the electron beam, thereby reducing free-electron laser gain and the accelerating gradient of the inverse free-electron laser (Esarey *et al.*, 1990 ▸; Yu *et al.*, 1992 ▸; Jia, 2002 ▸). A change in transverse velocity gives rise to a change in longitudinal velocity. This causes an error in phase between the electron and the ponderomotive wave and moves the electron away from the resonance. This kills the free-electron laser gain, and the accelerating gradient of the inverse free-electron laser drops below the tolerance level.

In this paper we address the issue of intrinsic type mis­alignment errors arising out of angular offsets between magnets in an undulator period. We study the misalignments through angular offsets at the clamping ends or random tilts at magnet–magnet joints. The random tilt of the magnets or poles generates undesirable magnet field components in both transverse and longitudinal directions. A strongly localized tilt gives rise to errors in period length and field amplitude. The localized errors are carried to the entire undulator segments and are a cause of concern for precision field integral and phase error measurements. In Section 2, we describe the design of a laser interferometer. It is shown that the interferometer can be used to read the offsets and fix the magnets to minimize the offsets. In Section 3, the results of measurements on a single magnet are presented and interferometer applications to control these types of intrinsic errors are discussed.

## 2. Laser interferometer

**Figure 2:**
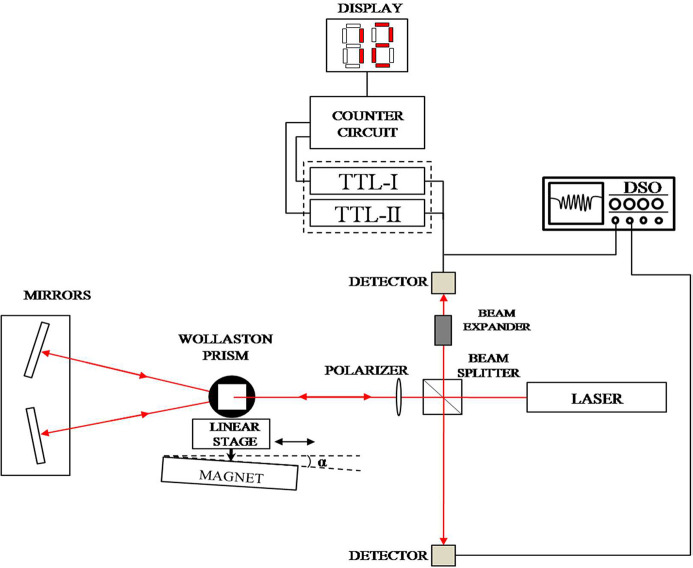
Schematic of the laser interferometer.

There is interest in the design and development of polarization interferometers for various applications in pure and applied sciences owing to their accuracy and stability (Wierzba & Kosmowski, 2005 ▸; Howard *et al.*, 2018 ▸; Hsieh *et al.*, 2018 ▸; Jiang *et al.*, 1996 ▸; Hatae *et al.*, 2009 ▸; Kemp *et al.*, 1998 ▸; Mishra *et al.*, 2020 ▸). Polarization interferometers with specific birefringent components and dedicated detection set-ups find application in molecular spectroscopy, astronomy, plasma diagnostic, fiber optics, optical metrology, oceanography, holography, remote sensing, and measurement of displacement, refractive index changes and surface irregularities. In the scheme, two coherent beams from a single source are required to produce interference by means of a birefringent optics. Specific birefringent optics such as polarization beam splitters, Wollaston prisms (WPs), polarisers, quarter-wave plates, and polarizing-maintaining fibers are essential components of polarization interferometers. Here we discuss another specific application of a WP-based polarization interferometer to remove intrinsic undulator errors. Fig. 2 ▸ illustrates the working principle of the laser interferometer. The light from the laser falls on the beam splitter. The light from the beam splitter continues to the WP through a polarizer. The light passing through the WP splits into two rays – the o-ray and the e-ray. The light is reflected back to the prism by the retro reflector. The reflected light rays from the retro reflector combine at the WP and pass through the polarizer and beam splitter and an interference fringe pattern is observed. The vertical displacements of the WP cause changes in the interference pattern which are used to measure the differences in the magnet height along the undulator magnet structure.

**Figure 3:**
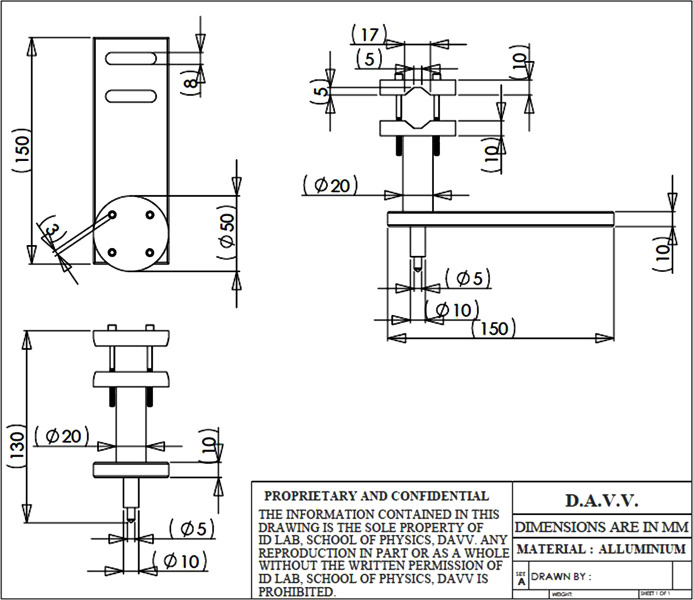
Design details of the WP and mirror holders.

In the experimental set-up, we use a 5 mW random polarization He–Ne laser at 632.8 nm (Thorlabs make, Model No. HNL050RB). The 1/*e* ^2^ beam diameter is 0.81 mm and the divergence is 1 mrad. The r.m.s. noise is less than 0.2%. The beam splitter (Newport, Model No. 10BC17MB) is a broad-band non-polarizing hybrid cube with moderate absorption and minimal polarization sensitivity. The beam splitter consists of a pair of precision right-angle prisms forming a 25.4 mm cube. The hypotenuse of one of the prisms is coated with a metal–dielectric hybrid coating which is optimized for transmission in the range 400–700 nm. The beam splitter material is Grade AN-BK7. The four faces are antireflection coated, providing less than 0.5% reflection over the 400–700 nm wavelength. The set-up uses a mounted calcite WP (Thorlabs, Model No. WP10-A). It has a beam separation angle of 20° at 633 nm and a clear aperture of 10 mm. The WP is held on a flat V-grooved holder, which is made up of two plates clamped by long screws. A groove in the lower and the upper plates helps to hold the WP tightly. The prism holder sits on a two co-axial cylindrical mechanical fixture, where an inner cylinder moves co-axially in and out in an outer cylinder of diameter 20 mm. The inner cylinder has a pointed end and moves smoothly over the magnet surface. The retro reflector is made up of two mirrors arranged on a rigid stand. The reflecting mirrors are mounted on kinematic mirror mounts (Holmarc India, Model No. KMR-CS-50). The silvered mirrors are 50 mm in length, 50 mm wide and 6 mm in thickness. The mirrors are fixed on rotating stages that provide suitable tilting angles as desired. The tilting angles are fixed at 10°. The system compatibility with the moving prism over long distances along the beam direction without obstructing the interference depends on the mirror size. For a mirror size of 50 mm, the distance is 294 mm (*d* = 50 mm/tan10°). Angular misalignment of the prism restricts the overlapping of the reflected spots from the mirror and the interference pattern disappears. A detailed drawing of the interferometer is shown in Fig. 3 ▸.

**Figure 4:**
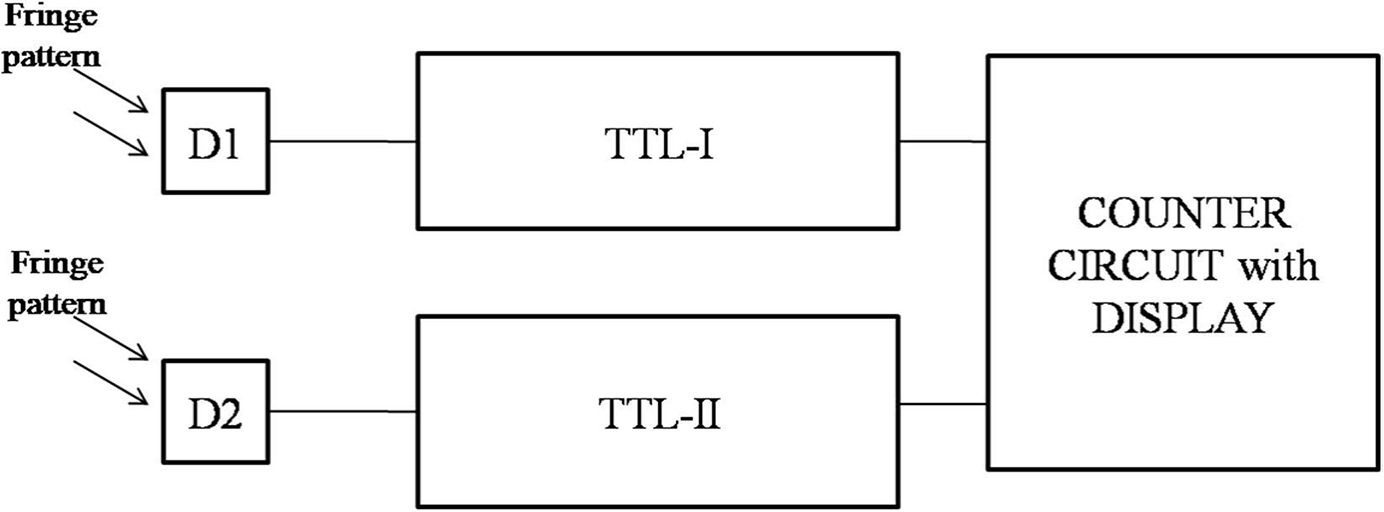
Block diagram of the fringe counter.

The detection system consists of an indigenously designed fringe counter and a digital oscilloscope connected to a computer. The fringe counter circuit consists of a photodiode as detector, a TTL (transistor logic) circuit having a current-to-voltage converter, amplifier circuits having inverting and non-inverting amplifiers, an LM311 integrated circuit (IC) to obtain TTL output, a D-latch and a counter circuit, as shown in Fig. 4 ▸. The detectors are placed in quadrature, *i.e.* a bright fringe falls on one detector and a half bright and half dark fringe falls on the other detector. As the WP of the interferometer moves, it causes a shift in the fringes. The output of the detectors goes to the TTL circuits and is then fed to the counter circuit to count the high–low transition of the TTL output and shows the fringe counting on the display.

**Figure 5:**
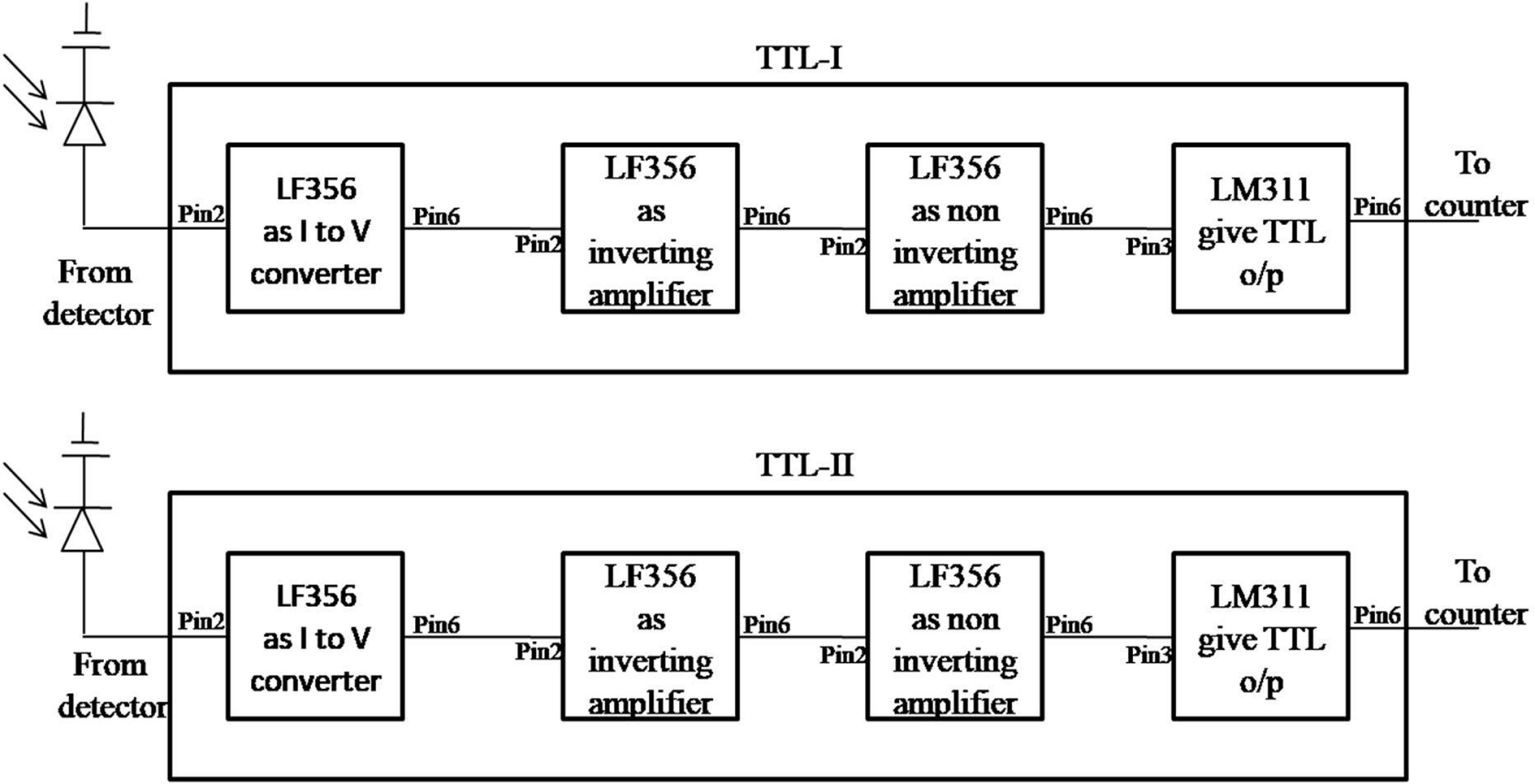
Block diagram of the TTL circuit.

**Figure 6:**
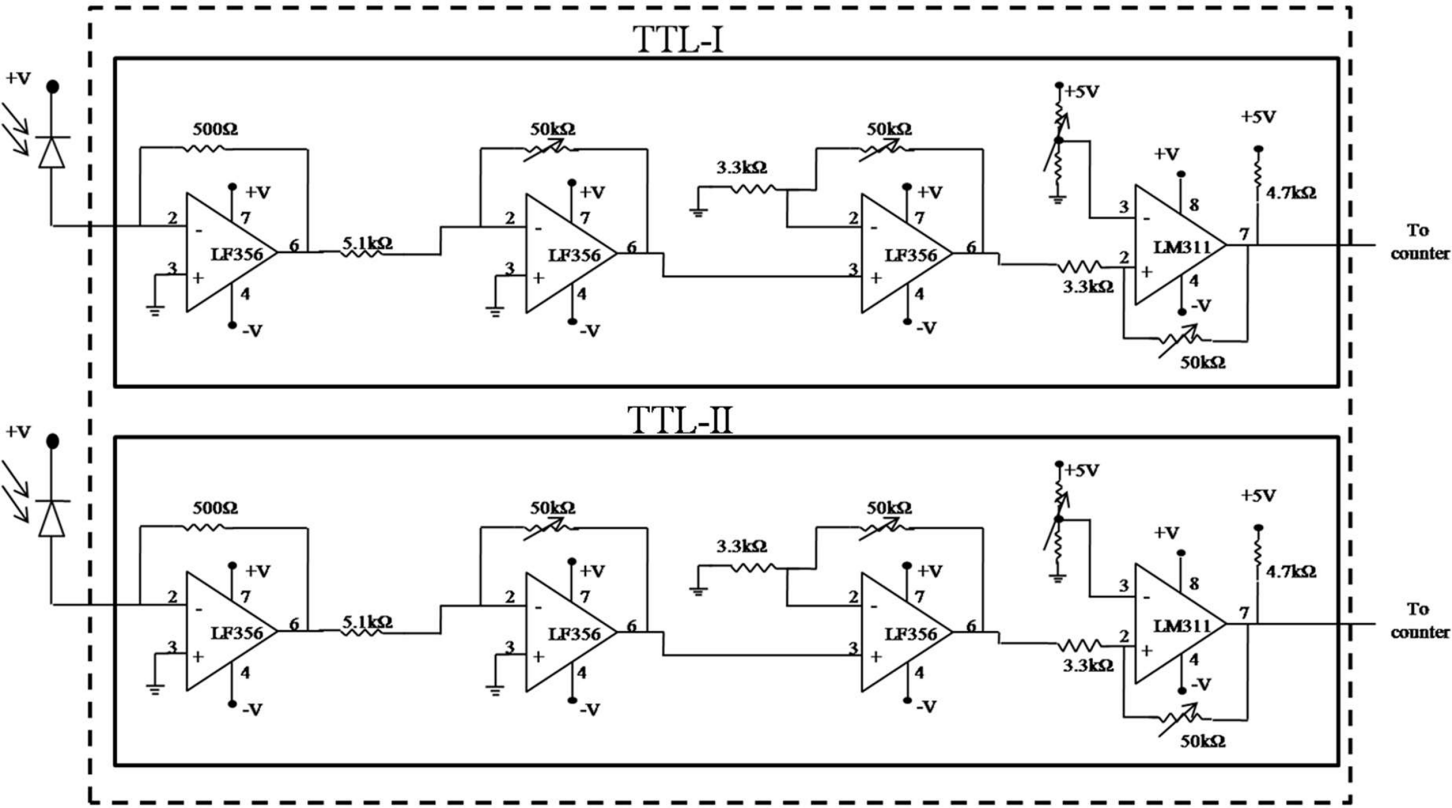
Circuit diagram of the TTL.

Block diagrams of TTL-I and TTL-II are shown in Fig. 5 ▸, and detailed circuit diagrams are shown in Fig. 6 ▸. The circuit contains a photodiode (model No. BPW34) operating in a reverse-bias condition which gives reverse current connected to the input of the current-to-voltage converter circuit made up of a six-pin IC LF356. The output from the photodiode is applied to the inverting terminal, *i.e.* Pin 2 of the IC. A 500 Ω resistor connected between Pin 2 and Pin 6 gives the feedback. Pin 1 and non-inverting terminal Pin 3 are grounded. Supply voltages to the IC of +15 V and −15 V are applied to Pin 7 and Pin 4, respectively. The output is taken from Pin 6 which is applied to the input of the inverting amplifier made up of IC LF356. An input resistance of 5.1 kΩ is connected to the inverting terminal, *i.e.* Pin 2. Pin 1 and non-inverting terminal Pin 3 are grounded. Supply voltages of +15 V and −15 V are connected to Pin 7 and Pin 4, respectively. A 50 kΩ variable-type feedback resistance three-pin trim pot whose two pins are shorted is connected between Pin 6 and Pin 2 by which the gain of the amplifier can be adjusted depending upon the input voltage. The output of the inverting amplifier is passed to the input of a non-inverting amplifier made up of IC LF356. Input is applied to non-inverting terminal Pin 3 of the IC. Supply voltages of +15 V and −15 V are connected to Pin 7 and Pin 4, respectively. Pin 1 is grounded and a 3.3 kΩ resistor is connected to inverting terminal Pin 2 and is grounded. A 50 kΩ variable-resistance trim pot whose two pins are shorted together is connected between Pin 6 and Pin 2; the gain can be adjusted. The output from this amplifier is taken from Pin 6 which is connected through a 3.3 kΩ resistor to input Pin 2 which is a non-inverting terminal of IC LM311. This IC provides TTL output, and supply voltages +15 V and −15 V are connected to Pin 8 and Pin 4, respectively. Pin 1 is grounded. The inverting terminal, *i.e.* Pin 3, is connected to a 10 kΩ variable-resistance three-pin trim pot whose first pin is connected to a +5 V supply, second pin is connected to Pin 3 of the IC, and the third pin is grounded. The value of this variable resistance is adjusted so that *V*_ref_ = 2 V is applied to Pin 3 of the IC. A +5 V is connected through a 4.7 kΩ resistor to Pin 7. The output from this circuit is taken from Pin 7. A trim pot of 50 kΩ is connected between Pin 2 and Pin 7 to adjust the hysteresis of the LM311 comparator circuit. There are two sets of the above circuit and the outputs of both circuits are passed on to the IC 7474 D-latch, one as input and the other as clock. Each supply voltage of +15 V and −15 V is bypassed with a 10 µf tantalum-type capacitor and a 0.1 µf ceramic-type capacitor to avoid ripples.

**Figure 7:**
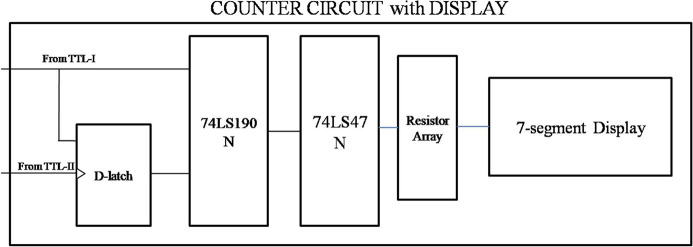
Block diagram of the counter circuit.

**Figure 8:**
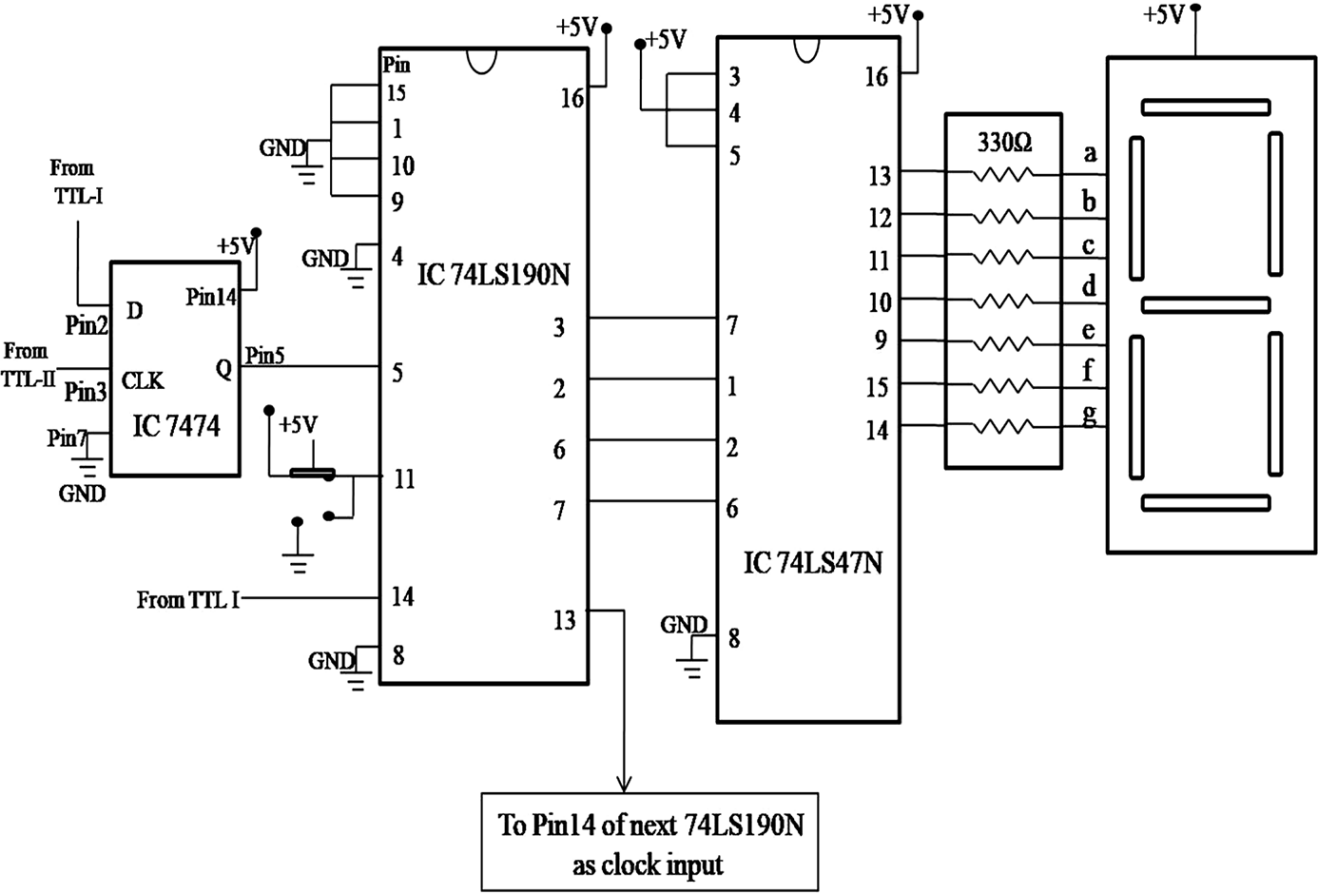
Circuit diagram of the counter with seven-segment display.

**Figure 9:**
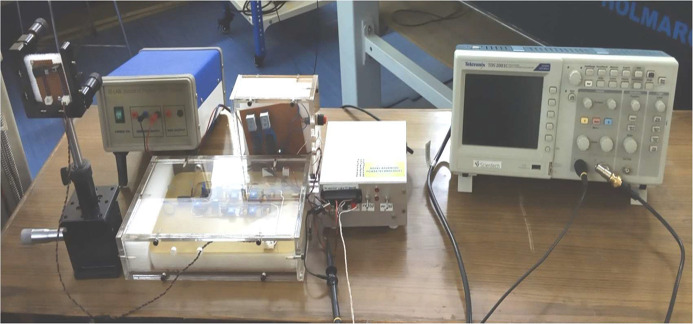
Detection system showing the fringe counter and digital oscilloscope.

A block diagram of the counter display system is shown in Fig. 7 ▸. The counter circuit shown in Fig. 8 ▸ contains IC 74LS190N, IC 74LS47N and a common anode seven-segment display. IC 74LS190N, having 16 pins and operating on a 5 V supply, counts the TTL output from IC LM311 and gives the output in binary which is then converted into decimal through IC 74LS47N, which shows output in the form of a number via a seven-segment display. Pin 16 of IC 74LS190N and IC 74LS47N are connected to a +5 V supply. Pins 15, 1, 10, 9 are connected to ground to present the counter value to 0. Pin 4 and Pin 8 are also connected to ground. Pin 11 is connected to +5 V and ground through a switch which toggles between them to count or reset the counter. Pin 5 is connected to the output of the D-latch as the clock to counter. Pin 3 is connected to Pin 7 of IC 74LS47N, Pin 2 to Pin 1, Pin 6 to Pin 2, and Pin 7 to Pin 6 of IC 74LS47N. Pins 3, 4, and 5 of IC 74LS47N are connected to +5 V. Pin 8 of IC 74LS47N is connected to ground and Pins 9 to 14 are connected to the seven-segment display through a resistor array having seven 330 Ω resistors to show the count number. This counter circuit is repeated by connecting Pin 13 of IC 74LS47N to Pin 14 as the clock of the next IC 74LS190N. A photograph of the detection system is given in Fig. 9 ▸.

## 3. Results and discussion

**Figure 10:**
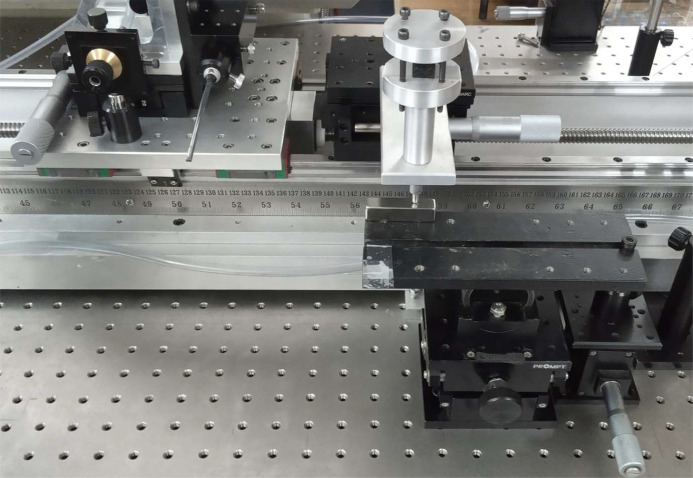
Magnet measurement set-up and interferometer readout.

**Figure 11:**
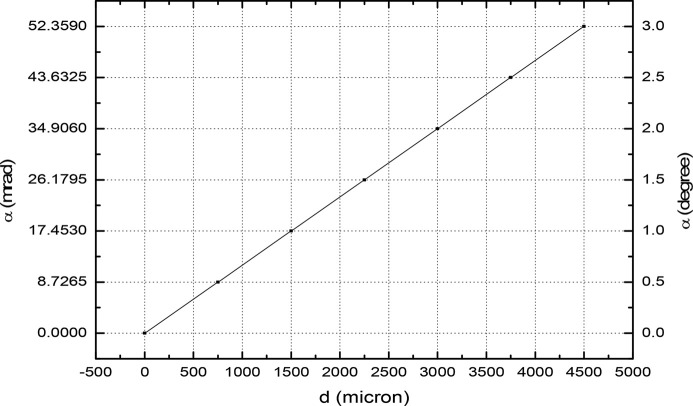
Calibration curve: angle versus displacement.

Fig. 10 ▸ shows the Hall probe measurement set-up on a single magnet. The magnet is kept on a plate which is given a tilt by means of a linear vertical stage attached to it. The calibration between the linear vertical stage displacement and angle is shown in Fig. 11 ▸. The slope of the curve calibration reads 12 µrad µm^−1^. The resolution of the vertical stage is 10 µm. For a 10 µm shift of the vertical stage we obtain a 6.8 millidegree angular offset. Here the readings are taken at a step length of 750 µm of the linear stage, which gives a 0.5° offset.

**Figure 12:**
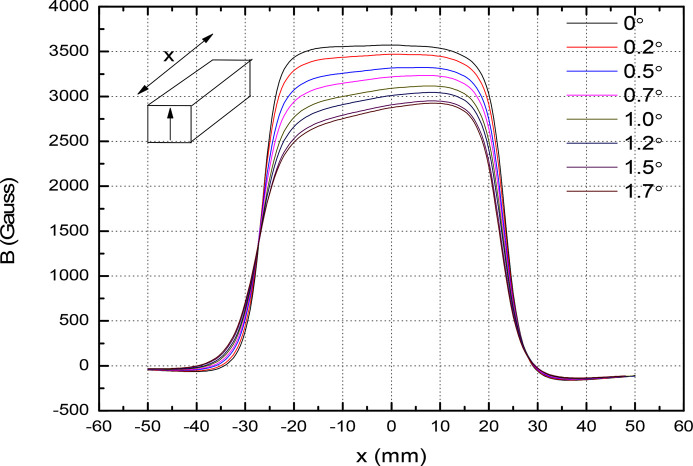
Effect of the angular tilt at the clamp end on the field along the horizontal direction.

**Figure 13:**
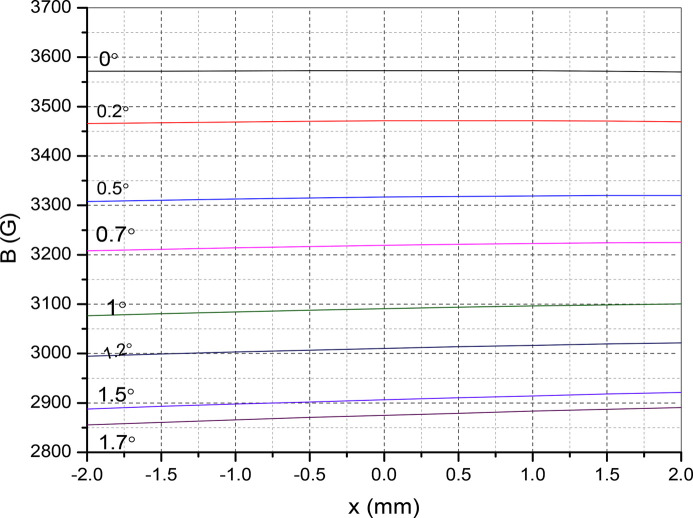
Effect of the angular tilt on the good field region.

**Figure 14:**
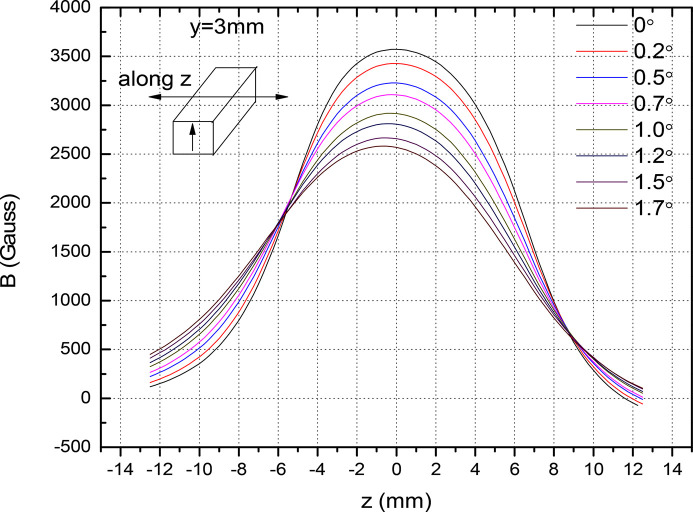
Effect of the angular tilt on the vertical field.

**Figure 15:**
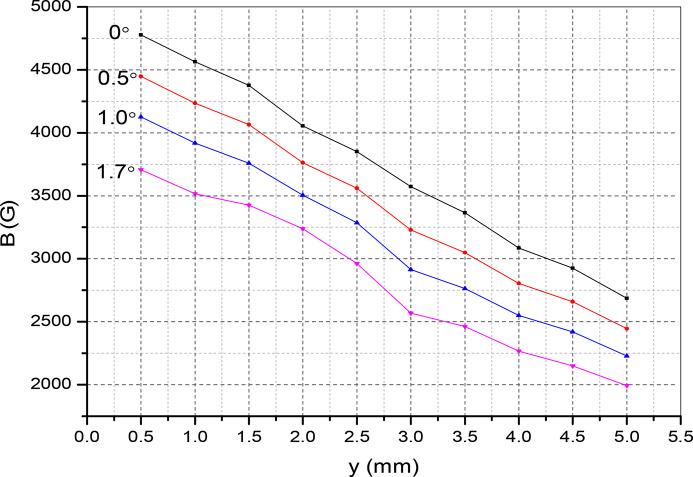
Effects of the tilt on the vertical field with distance away from the probe.

**Figure 16:**
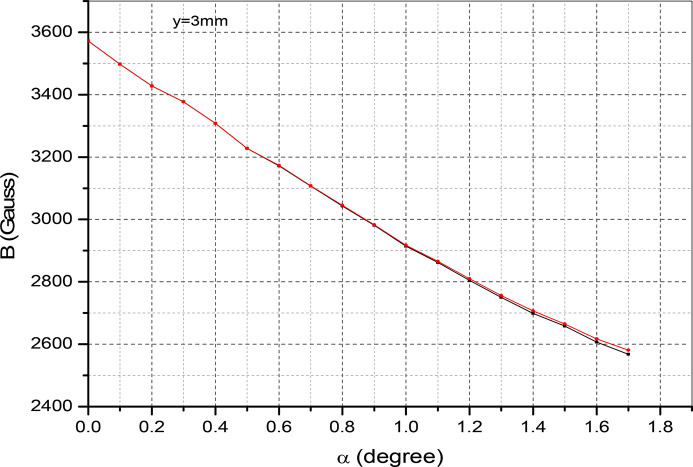
Effects of the tilt on the vertical field.

**Figure 17:**
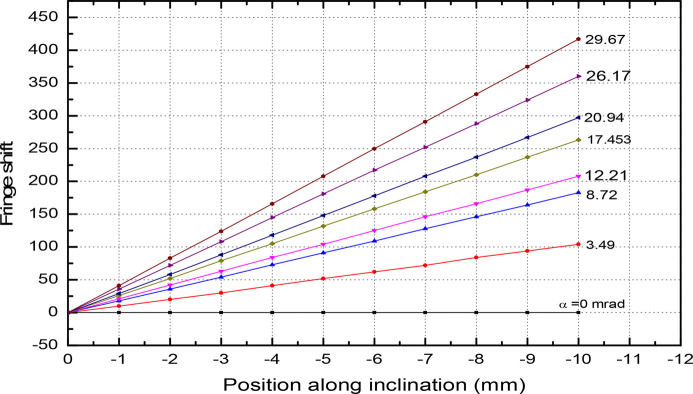
Fringe shift with different angle offset.

**Figure 18:**
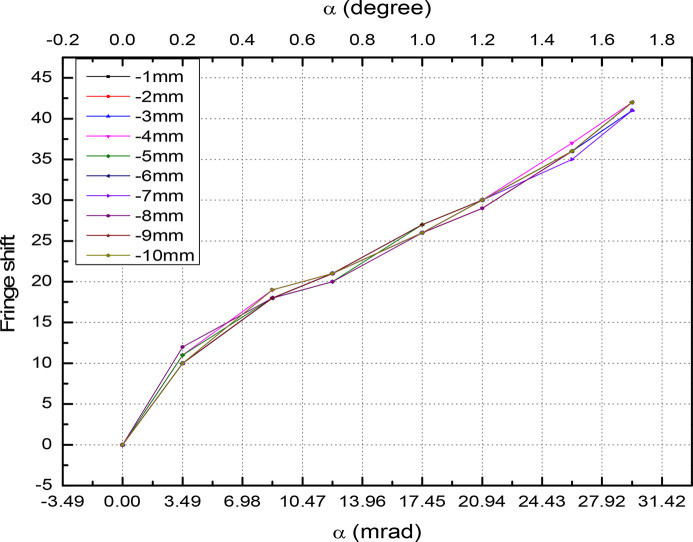
Fringe shift for several step lengths.

Fig. 12 ▸ represents the single magnet measurement in the horizontal direction. This type of angular offset arises if one end of the clamping is offset with respect to the other clamping end. In the figure, the tilting offset is shown as the negative side. The magnetic field lines deviate from the normal to the surface. A component of the oriented field is 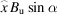 , where *B*_u_ is the field amplitude and α is the tilt angle. In the figure, the magnetic field drops to 3316 Gauss (*x* = 0, α = 0.5°) from 3572 Gauss (α = 0°, *x* = 0). This will affect the good field region of the magnet. This correspond to a 7% reduction. Fig. 13 ▸ shows the magnet field reduction in the region −2 mm < *x* < 2 mm. In Fig. 14 ▸ we show the effect of the angular offset along the longitudinal direction. The electron beam propagates along the longitudinal direction. A tilt α along the longitudinal direction offsets the magnetic field by 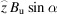 . This creates variation of the period length and fluctuations in the field amplitude along the beam direction. In Fig. 15 ▸ we plot the magnet field with probe movement along the vertical direction with and without tilting of the magnet. The field at α = 0°, *y* = 0.5 mm reads 4777 Gauss and drops to 4564 Gauss at α = 0°, *y* = 1 mm. This is about a 4.4% reduction in field amplitude. For a tilting angle of α = 1.0°, the field amplitude drops by 5%. In Fig. 16 ▸ the magnetic field reduction is plotted for several values of the tilting angle. The slope reads 581 Gauss per degree. The laser interferometer is rolled over the magnet surface to count the fringe shift – see Fig. 17 ▸. By reading out the fringe count, it is possible to correct the magnet tilt. For a 0.2° offset, the fringe count is 10 at a distance of 1 mm. The accuracy of the measurement is shown in Fig. 18 ▸. For several repeat measurements up to 1.7° angle offsets, the maximum fringe miss is one.

In conclusion, we have developed a laser interferometer to read the magnet misalignments in an undulator. The laser interferometer works on the basis of fringe shift. When the interferometer is rolled over the magnets, the fringe-shift count reads the magnet misalignments. A reduction in field amplitude is due to a split of the magnet field in components of an oriented magnetic field. A tilt of α° in a certain direction removes a field amplitude of *B*_u_sinα in that direction. A tilt along the horizontal direction decreases the good field region of the magnet. A tilt along the beam direction pushes the device to fluctuate in its period length and field amplitude. It is observed that the horizontal misalignment is more severe than the misalignment along the undulator length. This is because in a horizontal misalignment a part of the field component is removed away from the good field region of the undulator.
